# Anti-Fatigue Effects of Small Molecule Oligopeptides Isolated from *Panax ginseng* C. A. Meyer in Mice

**DOI:** 10.3390/nu8120807

**Published:** 2016-12-13

**Authors:** Lei Bao, Xiaxia Cai, Junbo Wang, Yuan Zhang, Bin Sun, Yong Li

**Affiliations:** 1 Department of Nutrition and Dietetics, Peking University International Hospital, Beijing 102206, China; baolei@pkuih.edu.cn; 2 Department of Nutrition and Food Hygiene, School of Public Health, Peking University, Beijing 100191, China; caixx1988@ccmu.edu.cn (X.C.); bmuwjbxy@bjmu.edu.cn (J.W.); zyzyzhangyuan@pku.edu.cn (Y.Z.); berseraphim@bjmu.edu.cn (B.S.); 3 School of Public Health, Beijing Key Laboratory of Environmental Toxicology, Capital Medical University, Beijing 100069, China

**Keywords:** ginseng oligopeptides, anti-fatigue, forced swimming test

## Abstract

*Panax ginseng* C. A. Meyer (ginseng) is an edible and medicinal Chinese herb, which is often used in Asian countries for physical fitness. Ginseng is reported to have a wide range of biological activity and pharmaceutical properties. There were more studies on ginsenosides and polysaccharides, but fewer studies on ginseng oligopeptides (GOP), which are small molecule oligopeptides isolated from ginseng. The present study was designed to evaluate the anti-fatigue effects of GOP in mice and explore the possible underlying mechanism. Mice were randomly divided into four experimental sets for the detection of different indicators. Each set of mice were then divided into four groups. The control group was administered distilled water, and three GOP intervention groups were administered 125, 250, and 500 mg/kg of body weight, respectively, of GOP by gavage each day. After 30 days of GOP treatment, it was observed that GOP could significantly increase the forced swimming time, enhance lactate dehydrogenase (LDH) activity and hepatic glycogen levels, and retard the accumulation of serum urea nitrogen (SUN) and blood lactic acid (BLA) in mice. GOP also markedly ameliorated fatigue-induced alterations of inoxidative stress biomarkers and antioxidant enzymes. Notably, GOP increased the mRNA expression of mitochondrial biogenesis factors and mitochondrial DNA content in skeletal muscles of mice. These results suggest that GOP possess anti-fatigue effects, which may be attributed to the inhibition of oxidative stress and the improvement of mitochondrial function in skeletal muscles. GOP could be a novel natural agent for relieving exercise fatigue.

## 1. Introduction

Fatigue is a feeling of extreme tiredness which can result in a broad range of physical and mental unfitness including inattention, distraction, and drowsiness [1,2]. It is mainly caused by the depletion of energy sources, which include the accumulation of end products of fatigue, the disorder of internal the environment, the decrease in glycemic levels and liver glycogen consumption [3]. Fatigue is a kind of sub-health status and may be associated with many illnesses. In addition, with an accelerating pace of life and fierce social competition, fatigue has become a common phenomenon. Thus, efforts to find a safe and effective method for the prevention of fatigue are necessary and significant.

It has been demonstrated that oxidative stress is one of the factors leading to fatigue [4]. High levels of oxidative stress lead to the excessive generation of reactive oxygen species (ROS) which are highly reactive molecules that cause lipid peroxidation of membrane structure and damage cellular structure. The release of ROS could result in lipid peroxidation of mitochondrial membrane. Damaged mitochondria was found to reduce cellular respiration and adenosine triphosphate (ATP) generation, and is suggested to be one of the primary causes of fatigue [5]. Interventions that reduce oxidative damage can effectively relieve fatigue, as some studies have shown that antioxidants have beneficial effects on fatigue [6,7]. The previous findings indicate that recovery from exercise-induced fatigue requires repairing the damage that has occurred in the body and/or prompting the elimination of metabolic products accumulated during exercise [8].

*Panax ginseng* C. A. Meyer (ginseng) is one of the traditional Chinese medicinal herbs that has been widely used for a long time [9]. Ginseng has extensive pharmacological functions including antitumoral, antioxidant, immune modulation, and normalizing the human metabolic system [10,11,12]. In traditional oriental medicine, ginseng has been mainly used to enhance stamina and to relieve physical stress and fatigue [13]. A randomized, double-blinded, placebo-controlled trial showed that *Panax ginseng* had anti-fatigue effects in patients with idiopathic chronic fatigue [14]. In addition, ginseng polysaccharides were also proven to have anti-fatigue activity in an animal study [15]. However, little is currently known about the anti-fatigue effects of ginseng oligopeptides (GOP), which are the general name for small molecule oligopeptides isolated from *Panax ginseng* C. A. Meyer. Thus, the present study was designed to evaluate the anti-fatigue activity of GOP and explore the possible underlying mechanism in mice.

## 2. Materials and Methods

### 2.1. Preparation and Identification of GOP

The GOP sample, which was derived from the roots of *Panax ginseng* C. A. Meyer planted in Jilin province, China, was provided by Jilin Taigu Biological Engineering Co., Ltd. (Jilin, China). In brief, ginseng roots were firstly boiled at 120–128 °C, 0.12–0.16 MPa for 0.5–1.5 h and then the concentrated solution was centrifuged. The supernatant was treated by enzymolysis with 5% special enzyme of ginseng for 3–5 h after adjusting the pH to 7.0 with sodium hydroxide. Then it was adsorbed with 3%–5% of active carbon, and the carbon was removed by filtration. After the filtrate was concentrated by vacuum concentration, the condensed liquid was dried by spray drying to obtain GOP powders.

The GOP sample was purified by HPLC (Waters Corporation, Milford, MA, USA) using a Phenomenex C18 column (10 mm × 250 mm), and the molecular weight distribution of the GOP sample was measured by LDI-1700 matrix-assisted laser desorption ionization time-of-flight mass spectrometry (MALDI-TOF-MS, Liner Scientific Inc., Reno, NV, USA). In addition, amino acid composition was further analyzed by an automatic amino acid analyzer (H835-50, Hitachi, Tokyo, Japan). The result showed that 95.42% of the GOP sample had a molecular weight between 180 and 1000 Da. Amino acids accounted for 3.94%, and the amino acid composition is shown in Table 1.

### 2.2. Chemicals and Reagents

Assay kits used for the determination of serum urea nitrogen (SUN) and lactate dehydrogenase (LDH) were purchased from Yingkexinchuang Science and Technology Ltd. (Macau, China). The detection kits of blood lactic acid (BLA), hepatic glycogen, superoxide dismutase (SOD), catalase (CAT), and malondialdehyde (MDA) were purchased from Nanjing Jiancheng Biotechnology Institute (Nanjing, China). All other reagents used in this study were of analytical grade.

### 2.3. Animals and Treatment

The present study, after approval from the Institutional Animal Care and Use Committee of Peking University (Ethical approval code: LA2015081, February 2015), used a total of 240 male ICR mice (6–8 weeks old, 18–22 g), which were procured from Animal Service of Health Science Center, Peking University. They were housed at 25 ± 1 °C, 50%–60% humidity, and maintained on a 12 h:12 h light-dark cycle, with free access to standard food and water. All animals were treated according to the Principles of Laboratory Animal Care (NIH publication No. 85-23, revised 1985) and the guidelines of Peking University Animal Research Committee.

After acclimatization for one week, the mice were randomly divided into four experimental sets (*n* = 60). Each set of mice were then divided into four groups (*n* = 15): control group (10 mL/kg distilled water), and three GOP intervention groups which were designated as a low-dose group (GOP-LG), medium-dose group (GOP-MG), and high-dose group (GOP-HG). GOP were accordingly administered to the mice of these three GOP intervention groups at 125, 250, and 500 mg/kg of body weight, respectively. The doses refer to the previous study in our lab [16]. Experimental mice were administrated by gavage for 30 days, and then were used for further experiments.

### 2.4. Forced Swimming Test

Mice from Experimental Set 1 were used for the forced swimming test. Forced swimming test was carried out as described previously [3]. Briefly, 30 minutes after the final treatments, the mice were placed individually in a swimming pool filled with water (25 ± 1 °C) to a depth of 30 cm with a lead sheath (5% of the mouse’s body weight) attached to the tail root of each mouse. The swimming time was recorded immediately when the physical strength of mouse was exhausted and it could not rise to the surface for more than 10 s.

### 2.5. Biochemical Assay

Mice from Experimental Set 2 were used for biochemical assay. Thirty minutes after the final oral administration, the mice were forced to swim in water at 30 °C for 90 min without any loads. After resting for an hour, a blood sample was obtained from the eyeballs and skeletal muscles (quadriceps femoris of both hind legs) of the mice. The serum was prepared by centrifugation at 2000 rpm at 4 °C for 15 min. The SUN content and LDH activity in serum were measured by automatic biochemical analyzer (Olympus Corporation, Tokyo, Japan). The SOD, CAT activity, and MDA levels in skeletal muscles were determined by detection kits according to the instructions.

### 2.6. Quantitative Real-Time PCR and Analyses of mtDNA Content

Total RNA and DNA were extracted from isolated skeletal muscles of mice from Experimental Set 2 by Trizol reagent (Invitrogen, Carlsbad, CA, USA) and DNeasy Tissue Kit (QIAGEN Sciences, Germantown, MD, USA), respectively. Real-time reverse transcription-PCR was performed using ABI 7300 real-time PCR detection system to detect the RNA expression of target genes with the specic primers:nuclear respiratory factor 1 (NRF-1), Forward 5′-CCATCTATCCGAAAGAGACAGC-3′ and Reverse 5′-GGGTGAGATGCAGAGTACAATC-3′; mitochondrial transcription factor A (TFAM), Forward 5′-CCTGAGGAAAAGCAGGCATA-3′ and Reverse 5′-TCACTTCGTCCAACTTCAGC-3′; β-actin, Forward 5′-GATTACTGCTCTGGCTCCTAGC -3′ and Reverse 5′-GACTCATCGTACTCCTGCTTGC-3′. Real-time PCR was used to detect mtDNA copy number. The sequences are as follows: Mitochondrial DNA (mtDNA), Forward 5′-CGTTAGGTCAAGGTGTAGCC-3′ and Reverse 5′-CCAGA CACACTTTCCAGTATG-3′; β-actin, Forward 5′-GATTACTGCTCTGGCTCCTAG C-3′ and Reverse 5′-GACTCATCGTACTCCTGCTTGC-3′. Cycling conditions were 95 °C for 5 min followed by 40 repeats of 95 °C for 10 s and 60 °C for 30 s. Target mRNA values and the content of mtDNA copy number were determined by comparison to the control sample after being normalized to β-actin levels and calculated using the comparative cycle threshold (^∆∆^*Ct*) method.

### 2.7. Determination of Blood Lactic Acid

The concentrations of BLA were determined in mice from Experimental Set 3. Thirty minutes after the final oral administration, the mice were forced to swim in water at 30 °C for 10 min without any loads. Blood was obtained at three time points: at baseline, 0 min after swimming, and 20 min after swimming. 20 μL blood was accurately collected from the angular vein of mice by glass capillary each time and then immediately moved into the bottom of a 5 mL centrifuge tube which was joined with 0.48 ml 1% sodium fluoride solution in advance. The glass capillary was flushed with supernatant several times. The concentrations of BLA were determined according to the procedures provided by the kits. The area under the BLA curve (AUC) was calculated according to the following formula (1).

Cs = 1/2 × (C_0_ + C_1_) × 10 + 1/2 × (C_1_ + C_2_) × 20 (1)

C_0_, C_1_, and C_2_ stand for the BLA concentration of mice at baseline, 0 and 20 min after swimming, respectively. Cs stands for the area under the BLA curve.

### 2.8. Examination of Hepatic Glycogen

Mice from Experimental Set 4 were used to examine hepatic glycogen. Thirty minutes after the last administration of GOP, The mice were killed and their livers were immediately isolated and homogenized to 10% solution with normal saline at 4 °C. Hepatic glycogen levels were determined using available kits.

### 2.9. Statistical Analysis

The data were expressed as mean ± standard deviation (SD). Differences between groups were analyzed by one-way ANOVA test followed by Tukey’s post hoc least significant difference test if variances were equal or Tamhane’s T3 test if variances were not equal. *p* < 0.05 was considered significant.

## 3. Results

### 3.1. Effects of GOP on the Body Weight of Mice

The effects of GOP on the body weight of mice during the experiment are shown in Table 2. The results showed that there was no statistical significance among the groups in Experimental Set 1, 2, 3 and 4, respectively.

### 3.2. Effects of GOP in the Forced Swimming Test

**Figure 1:**
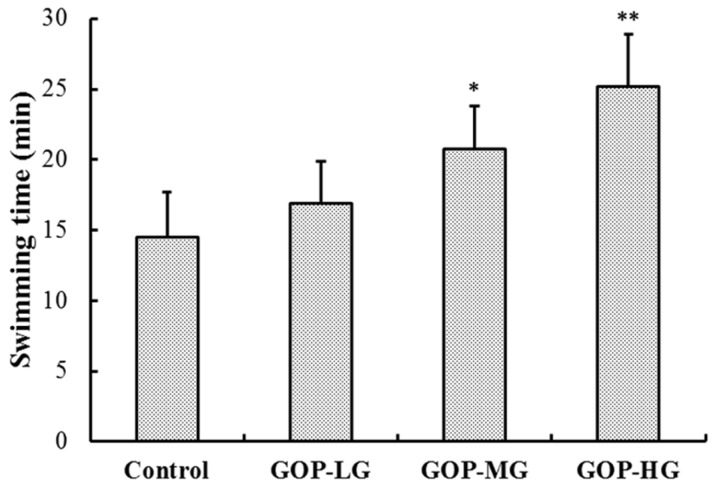
Effects of GOP on the forced swimming time in mice. Data were presented as means ± SD (*n* = 15). * *p* < 0.05, ** *p* < 0.01 versus control group. GOP-LG, ginseng oligopeptides low-dose group; GOP-MG, ginseng oligopeptides medium-dose group; GOP-HG, ginseng oligopeptides high-dose group.

The effects of GOP on the forced swimming time of mice are shown in Figure 1. As expected, in comparison with the control group, the forced swimming time in all three GOP groups was longer and the difference was statistically significant in the medium-dose GOP group (GOP-MG) and high-dose GOP group (GOP-HG) (*p* < 0.05 and *p* < 0.01). In general, when compared to the control group, the forced swimming time in the low-dose GOP group (GOP-LG), GOP-MG, and GOP-HG increased by 16.5%, 42.7%, and 73.7%, respectively.

### 3.3. Effects of GOP on Lactate Dehydrogenase (LDH), Serum Urea Nitrogen (SUN) and Hepatic Glycogen Content in Mice

**Figure 2:**
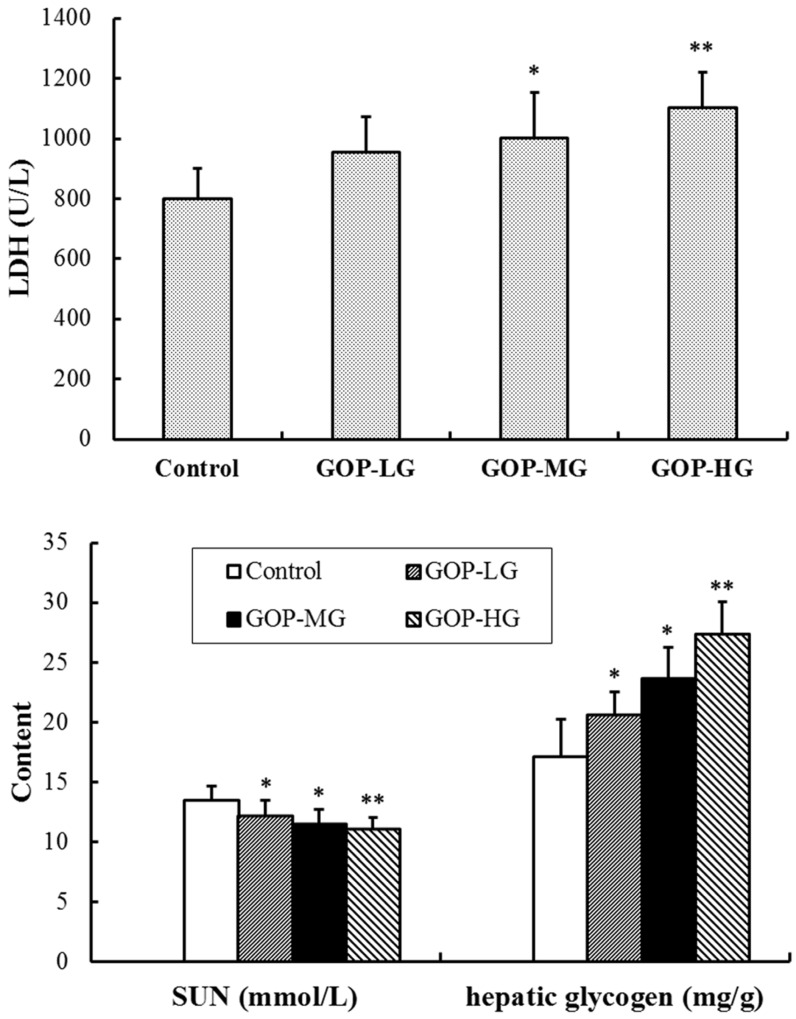
Effects of GOP on lactate dehydrogenase (LDH), serum urea nitrogen (SUN), and hepatic glycogen content in mice. Data were presented as means ± SD (*n* = 15). * *p* < 0.05, ** *p* < 0.01 versus control group. GOP-LG, ginseng oligopeptides low-dose group; GOP-MG, ginseng oligopeptides medium-dose group; GOP-HG, ginseng oligopeptides high-dose group.

As shown in Figure 2, compared with the control group, the LDH activity was significantly increased in GOP-MG and GOP-HG (*p* < 0.05 and *p* < 0.01) and the SUN levels were markedly decreased in all three GOP groups (*p* < 0.05 for GOP-LG, *p* < 0.05 for GOP-MG and *p* < 0.01 for GOP-HG) by GOP treatment after swimming. Moreover, the hepatic glycogen levels of mice were markedly improved in GOP-LG, GOP-MG, and GOP-HG (*p* < 0.05, *p* < 0.05, and *p* < 0.01).

### 3.4. Effects of GOP on Blood Lactic Acid (BLA) Levels in Mice

The results about the effects of GOP on BLA in mice at different time points are shown in Table 3. There were no significant differences among the groups at baseline. Compared with the control group, the concentrations of BLA in GOP-MG and GOP-HG were significantly decreased at 0 min after swimming (*p* < 0.05 and *p* < 0.01). At 20 min after swimming, the concentrations of BLA in all three GOP groups were significantly decreased (*p* < 0.05 for GOP-LG, *p* < 0.01 for GOP-MG and *p* < 0.01 for GOP-HG). After GOP treatment, the area under the BLA curve (AUC) was also reduced in comparison with the control group (*p* < 0.05 for GOP-LG, *p* < 0.05 for GOP-MG, and *p* < 0.01 for GOP-HG).

### 3.5. Effects of GOP on Parameters of Oxidative Stress in Skeletal Muscles of Mice

The SOD, CAT activity, and MDA levels are shown in Table 4 to evaluate the level of oxidative stress in skeletal muscles of mice. After the treatment, the activity of SOD and CAT was significantly improved in GOP-MG and GOP-HG (*p* < 0.05 and *p* < 0.01). In addition, MDA levels in skeletal muscle were significantly attenuated in GOP groups (*p* < 0.05 for GOP-LG, *p* < 0.05 for GOP-MG and *p* < 0.01 for GOP-HG) compared with the control group.

### 3.6. Effect of GOP on Mitochondrial Biogenesis Factors and mtDNA Content in Skeletal Muscles of Mice

**Figure 3:**
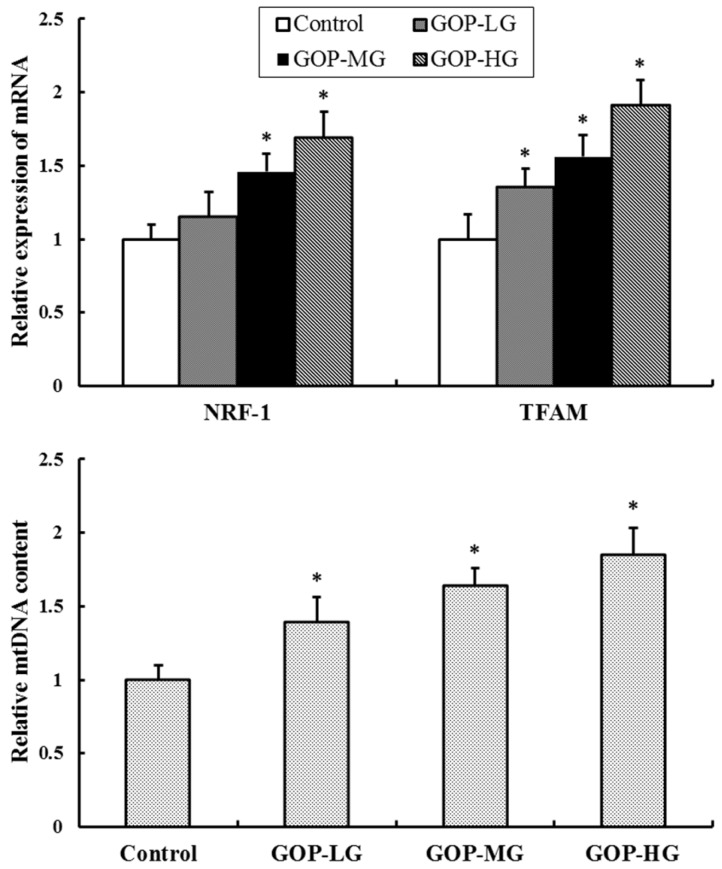
Effects of GOP on the RNA expression of nuclear respiratory factor 1 (NRF-1), Mitochondrial transcription factor A (TFAM) and mitochondrial DNA (mtDNA) copy number in skeletal muscles of mice by real-time PCR analysis. β-actin mRNA levels were used as a control. Values were represented as means ± SD. * *p* < 0.05 versus control group. GOP-LG, ginseng oligopeptides low-dose group; GOP-MG, ginseng oligopeptides medium-dose group; GOP-HG, ginseng oligopeptides high-dose group

As shown in Figure 3, the mRNA expression of NRF-1 and TFAM, which were mitochondrial biogenesis factors considered essential for mitochondrial gene expression in mammals, was markedly increased in GOP groups compared with the control group (*p* < 0.05). In addition, the mtDNA content was also significantly improved after the GOP treatment (*p* < 0.05 for each).

## 4. Discussion

Ginseng has several pharmaceutical functions, such as antitumoral, antioxidant, and hypoglycemic properties [17,18]. In Asian countries, relieving fatigue is one of its traditional uses [19]. Ginseng contains a variety of active constituents such as ginsenosides, polysaccharides, peptides, polyacetylenes, and phenolic compounds. Much attention has been focused on the bioactive components of saponins and polysaccharides presented in ginseng. However, the effects of GOP, which include small molecule oligopeptides isolated from *Panax ginseng* C. A. Meyer, have not yet been investigated. In the present study, we found that GOP could increase the forced swimming time, LDH activity, and the hepatic glycogen levels, and simulataneously decrease the contents of SUN and BLA in mice. The anti-fatigue effect may be associated with the inhibition of oxidative stress and the improvement of mitochondrial activity.

Repetitive and sustained physical labor results in a fatigued condition, which can provoke a series of systematic alterations including endocrine, immune, and metabolic dysfunction [20]. The forced swimming test is a good experimental model for evaluating the capacity of anti-fatigue in mice [21]. In the present study, we found GOP treatment prolonged the exhausting time of mice, especially at 250 and 500 mg/kg of body weight, indicating the anti-fatigue effects of GOP on mice. To further study the anti-fatigue property of GOP, several biochemical markers for fatigue were measured, including SUN, LDH, BLA, and hepatic glycogen. SUN, formed in the liver as a metabolic product of protein and amino acid, is one of blood biochemical indexes related to fatigue. Along with the increasing quantity of exercise, the energy from sugar and fat catabolism is not sufficient for the body, and then proteins and amino acids have a stronger catabolism to compensate for the energy consumption, which causes a rise in SUN [22]. There was a remarkable positive correlation between the level of SUN and degree of fatigue [23]. In the process of long-lasting exercise, excess lactic acid is generated and accumulated in skeletal muscles, which is the major cause of muscle fatigue [24]. Therefore, BLA can be used as an index of fatigue. In addition, glycogen is an important energy material for movement and provides enough energy for muscle contraction. Energy use brings on the reduction of glycogen and an increase in hepatic glycogen will improve exercise endurance [25]. In the present study, GOP could increase the LDH activity and the hepatic glycogen levels, and decrease the contents of SUN and BLA in mice. These findings are consistent with the previous studies, which reported similar results in other ginseng extracts, such as proteins and water-soluble polysaccharides [15,26].

High consumption of energy during intense exercise may cause an imbalance between the oxidation and anti-oxidation systems, causing an increase of ROS and a reduction of antioxidant activities, subsequently leading to the increased production of ROS. Oxidative stress has been proven to be implicated in both chronic fatigue and fatigue-related disorders [27]. Extreme physical stress could lead to excess generation of ROS in the skeletal muscle which, in turn, results in peripheral fatigue [28,29]. To evaluate the antioxidant ability of GOP, SOD, CAT activity, and MDA levels were measured, which are generally considered indicators of the capacity of the antioxidant defensive system. SOD and CAT are important enzymatic antioxidant systems for scavenging free radicals and their metabolites [30]. MDA is one of the degradation products of lipid peroxidation, which is an important indicator for evaluating cellular oxidative stress [31]. Animal and human studies showed that *Panax ginseng* has remarkable anti-oxidative actions [14,32,33]. Our results suggested that the anti-fatigue effects of GOP are closely related to the protection of corpuscular membrane by improving activities of several enzymes and preventing lipid oxidation.

One of the interesting findings in the present study was that mitochondrial function was improved in skeletal muscles of mice after GOP treatment. It is known that continuous ATP generation is required in myocytes to maintain prolonged physical activity. The mitochondrion is an important intracellular organelle in eukaryotic cells, which is the main venue of oxidative phosphorylation and ATP production in mammal cells. Moreover, the mitochondrion plays an important mediating role for oxidative stress [34]. Consequently, mitochondrial function in skeletal muscles contributes to exercise-induced fatigue. In this study, NRF-1, TFAM, and mtDNA copy numbers were measured to evaluate mitochondrial function. NRF-1 is a positive regulator of transcription, which initiates the synthesis of mitochondrial proteins, including mitochondrial import proteins, heme biosynthesis proteins, components of the electron transport chain complexes, cytochrome c, and TFAM [35]. TFAM is also a key transcription factor for the regulation of mitochondrial gene transcription and a direct regulator of mtDNA duplication [36]. MtDNA copy number was considered as a surrogate marker of mitochondrial function [37]. Liu et al. demonstrated that ginsenoside Rd, one of the active components of *Panax ginseng*, was able to reduce intracellular ROS levels, enhance antioxidant enzymatic activities, stabilize the mitochondrial membrane potential, and increase intracellular ATP levels [38]. Kim et al. also found that ginsenoside Rg3 increased ATP levels in C2C12 myotubes and enhanced the expression of key genes involved in mitochondrial biogenesis such as NRF-1, TFAM, and peroxisome proliferator-activated receptor γ coactivator 1α (PGC-1α) which is a key regulator that induces mitochondrial biogenesis by activating other transcription factors [39]. In the present study, we found GOP could improve mitochondrial function in skeletal muscles of mice by restoring the mtDNA content and increasing the mRNA expression of NRF-1 and TFAM, thereby suppressing oxidative stress and generating more ATP for energy supplement. This might be a potential mechanism of the anti-fatigue effects of GOP in mice. In the present study, the suitable dose of GOP is 250–500 mg/kg of body weight in mice. There are currently few studies about GOP in human. A preliminary report declared that the regular dosage of ginseng was under 1 g/day [40]. However, a randomized, double-blinded, placebo-controlled trial showed that administering 2 g/day of ginseng extract was beneficial to patients with idiopathic chronic fatigue [14]. Further studies are needed to explore the optimal dose of GOP to generate their anti-fatigue effects in humans.

## 5. Conclusions

Taken together, our results showed for the first time that GOP possess anti-fatigue effects. GOP could increase the forced swimming time of mice by enhancing LDH activity and hepatic glycogen levels, and retarding the accumulation of SUN and BLA. Moreover, GOP could improve mitochondrial function and inhibit oxidative stress in skeletal muscles of mice, which may be an action pathway of its anti-fatigue effects. GOP could be a novel natural agent for exercise fatigue. Further research in vitro will be required to explore the exact molecular mechanism by which GOP play their role in anti-fatigue effects.

## Figures and Tables

**Table 1:** Amino acid composition of GOP.

Amino Acid	Amino Acid Composition Of GOP (g/100 g)
Asp	0.19
Glu	0.12
Ser	0.02
His	0.06
Gly	0.02
Thr	0.05
Arg	2.26
Ala	0.13
Tyr	0.09
Cys	0.01
Val	0.06
Met	0.02
Phe	0.09
Ile	0.04
Leu	0.08
Lys	0.06
Pro	0.65

GOP: ginseng oligopeptides.

**Table 2:** Effects of GOP on the body weight in mice.

Body Weight (g)	Control	GOP-LG	GOP-MG	GOP-HG
Set 1				
Initial body weight	25.22 ± 1.34	25.50 ± 1.12	25.41 ± 0.93	25.61 ± 1.31
Terminal weight	34.85 ± 2.65	34.39 ± 2.99	34.50 ± 2.05	35.83 ± 1.75
Set 2				
Initial body weight	25.33 ± 1.43	25.86 ± 1.35	25.76 ± 1.35	25.90 ± 1.13
Terminal weight	36.65 ± 3.63	35.73 ± 2.87	35.28 ± 3.14	35.47 ± 2.43
Set 3				
Initial body weight	25.53 ± 1.64	25.89 ± 1.42	25.34 ± 1.38	25.87 ± 1.32
Terminal weight	36.28 ± 3.07	36.81 ± 3.13	36.75 ± 2.79	36.42 ± 3.26
Set 4				
Initial body weight	24.98 ± 1.18	25.55 ± 1.07	24.83 ± 1.27	24.65 ± 1.15
Terminal weight	35.47 ± 2.73	35.03 ± 3.05	35.58 ± 2.73	35.04 ± 2.12

Data are expressed as means ± SD; *n* = 15 for each group. GOP-LG, ginseng oligopeptides low-dose group; GOP-MG, ginseng oligopeptides medium-dose group; GOP-HG, ginseng oligopeptides high-dose group.

**Table 3:** Effects of GOP on the content of BLA at different time points in mice.

BLA (mg/L)	Control	GOP-LG	GOP-MG	GOP-HG
Baseline	195.22 ± 38.43	192.50 ± 41.92	188.46 ± 33.66	195.83 ± 32.31
0 min after swimming	437.59 ± 42.56	384.03 ± 42.49	354.05 ± 39.09 *	355.83 ± 31.55 **
20 min after swimming	345.23 ± 41.83	275.06 ± 49.05 *	265.07 ± 41.35 **	247.91 ± 34.26 **
Area under BLA curve	10,997.62 ± 998.71	9475.45 ± 930.05 *	8651.26 ± 901.64 *	8457.56 ± 843.35 **

Data are expressed as means ± SD; *n* = 15 for each group. BLA, blood lactate. * *p* < 0.05, ** *p* < 0.01 versus control group. GOP-LG, ginseng oligopeptides low-dose group; GOP-MG, ginseng oligopeptides medium-dose group; GOP-HG, ginseng oligopeptides high-dose group.

**Table 4:** Effects of GOP on SOD, CAT activity, and MDA levels in skeletal muscles of mice.

Parameters	Control	GOP-LG	GOP-MG	GOP-HG
SOD (U/mg·pro)	96.10 ± 9.05	105.19 ± 10.98	115.91 ± 10.30 *	123.69 ± 11.59 **
CAT (U/mg·pro)	95.86 ± 15.23	111.77 ± 19.43	122.46 ± 11.36 *	129.37 ± 13.92 **
MDA (nmol/mg·pro)	6.59 ± 0.26	6.04 ± 0.29 *	5.97 ± 0.19 *	5.41 ± 0.23 **

Data are expressed as means ± SD; *n* = 15 for each group. SOD, superoxide dismutase; CAT, catalase; MDA, malondialdehyde. * *p* < 0.05, ** *p* < 0.01 versus control group. GOP-LG, ginseng oligopeptides low-dose group; GOP-MG, ginseng oligopeptides medium-dose group; GOP-HG, ginseng oligopeptides high-dose group.

## Abbreviations

ATP	Adenosine Triphosphate
BLA	Blood Lactic Acid
CAT	Catalase
GOP	Ginseng Oligopeptides
LDH	Lactate Dehydrogenase
MDA	Malondialdehyde
mtDNA	Mitochondrial DNA
NRF-1	Nuclear Respiratory Factor 1
ROS	Reactive Oxygen Species
SOD	Superoxide Dismutase
SUN	Serum Urea Nitrogen
TFAM	Mitochondrial Transcription Factor A
